# MiR-29a deficiency disrupts gut microbiota-bile acid homeostasis and impairs the anti-inflammatory effects of lithocholic acid in colitis

**DOI:** 10.3389/fcimb.2026.1867553

**Published:** 2026-09-17

**Authors:** Xi Xiao, Qian Gong, Xuexia Zheng, Junhao Zhou, Cui Shao, Qian Zuo, Chenhao Cai

**Affiliations:** 1 The First School of Clinical Medicine, Guangzhou University of Chinese Medicine, Guangzhou, China; 2 Postdoctoral Research Station, Guangzhou University of Chinese Medicine, Guangzhou, China; 3 School of Traditional Chinese Medicine (TCM) and Rehabilitation Science, Guangdong Chaozhou Health Vocational College, Chaozhou, China; 4 Wingngaiyuen Chinese Medicine Clinic, Hong Kong, Hong Kong SAR, China; 5 Department of Oncology, The Affiliated Traditional Chinese Medicine (TCM) Hospital of Guangzhou Medical University, Guangzhou Medical University, Guangzhou, China; 6 Department of Breast, The Second Affiliated Hospital of Guangzhou University of Chinese Medicine, Guangzhou, China

**Keywords:** bile acid metabolism, colitis, gut microbiota, host-microbiota interaction, intestinal inflammation, lithocholic acid, miR-29a

## Abstract

**Background:**

Gut microbiota, bile acid metabolism, and their derived metabolites are critical regulators of intestinal inflammation in inflammatory bowel disease (IBD). While microRNA-29a (miR-29a) is a recognized epigenetic regulator of intestinal inflammation, its role in shaping microbiota-driven bile acid metabolism, particularly secondary bile acid production, remains unclear. This study aimed to investigate how miR-29a regulates microbiota-bile acid axis, with an emphasis on lithocholic acid (LCA) and its anti-inflammatory effects in colitis.

**Methods:**

Colitis was induced in wild-type and *Mir29a/Mir29b-1* locus-deficient mice using dextran sulfate sodium (DSS), and disease severity, colonic injury, and inflammatory responses were assessed. Fecal microbiota composition and bile acid profiles were characterized using 16S rRNA gene sequencing and targeted metabolomics. Alterations in bile acids were further examined in relation to disease severity. *In vitro*, Caco-2 cells were used to evaluate the anti-inflammatory effects of LCA under modulation of miR-29a expression.

**Results:**

MiR-29a/b1 deficiency significantly exacerbated DSS-induced disease severity, colonic injury, inflammatory responses, and epithelial ultrastructural damage. KO-DSS mice exhibited gut microbiota dysbiosis, with reduced microbial diversity and altered community structure. Targeted metabolomics revealed impaired primary-to-secondary BA biotransformation, characterized by chenodeoxycholic acid (CDCA) accumulation and a reduced LCA/CDCA ratio. *In vitro*, LCA supplementation attenuated DSS-induced pro-inflammatory gene expression, whereas this protective effect was partially diminished by miR-29a inhibition.

**Conclusions:**

These results support a role for miR-29a in maintaining gut microbial bile acid homeostasis during colitis and contributing to the epithelial anti-inflammatory response to LCA. Together, these findings link host microRNA regulation to microbiota-derived BA function and may inform future precision strategies for IBD.

## 1 Introduction

Inflammatory bowel disease (IBD) represents an increasing global health burden characterized by distinct epidemiological transitions, including disease emergence, rising incidence, increasing cumulative prevalence, and eventual stabilization of prevalence. Many highly industrialized nations are presently experiencing a sustained increase in the number of individuals living with IBD and are expected to approach a prevalence of more than 1% of the population over the coming decade, with global IBD population projected to exceed 10 million patients by 2030 (Kaplan and Windsor, 2021; Kaplan, 2025). Over the past decade, IBD has increasingly been recognized as a multifactorial disorder shaped not only by genetic susceptibility and host immune disruption, but also by nutritional influences, environmental exposures, and microbial-metabolic alterations (Kong et al., 2023). Traditionally, IBD treatment relied on corticosteroids, immunosuppressants, and 5-aminosalicylates (Baumgart and Sandborn, 2007). These treatments, however, have various drawbacks, notably high costs, adverse reactions, and reduced effectiveness during treatment; initial non-response occurs in 30-40% of cases, while approximately 13% of patients experiencing a secondary loss of response each year (Appiah et al., 2025). Therefore, novel therapeutic strategies are urgently needed.

Numerous studies to date have stated that addressing the gut microbiota-bile acid (BA) axis could be a key strategy in treating IBD (Pan et al., 2024). Among the metabolic disturbances characteristic of IBD, disruption of BA homeostasis has emerged as a pivotal pathological feature (Thomas et al., 2022). IBD patients consistently exhibit elevated primary BA concentrations alongside markedly reduced secondary BA levels, owing to diminished populations of BA-metabolizing bacteria (Yang et al., 2021). Within this altered metabolic landscape, lithocholic acid (LCA), a bioactive secondary BA generated from chenodeoxycholic acid through microbial transformation, stands out for its capacity to suppress epithelial cytokine release and reduce mucosal inflammation, with microbial production contributing to its anti-inflammatory activity (Rochat et al., 2007; Mohamadzadeh et al., 2011; Thomas et al., 2022). In intestinal inflammatory models, one reported mechanism involves LCA-mediated activation of pregnane X receptor (PXR), with subsequent suppression of TLR4-mediated NF-κB/NLRP3 inflammasome signaling, thereby attenuating colonic inflammation and restoring intestinal barrier function (Huang et al., 2018). These findings highlight LCA as a microbiota-derived metabolite with potential therapeutic relevance in IBD-associated inflammation.

Recent studies indicate that microRNAs (miRNAs) function as critical regulators of BA metabolism, influencing hepatic BA synthesis, uptake, and secretion through the post-transcriptional modulation of CYP7A1 and CYP8B1. Mechanistically, miR-122a and miR-422a serve as negative regulators of BA synthesis through direct binding to the 3’-UTRs of CYP7A1 and CYP8B1 (Song et al., 2010). Beyond direct targeting, certain miRNAs regulate BA synthesis through broader regulatory networks. For example, miR-33a coordinates with SREBP2 to co-regulate lipid and BA homeostasis (Rayner et al., 2010), while miR-24 and miR-34a exert indirect suppressive effects by modulating the hepatic transcription factor HNF4α (Salloum-Asfar et al., 2016). These insights indicate a synergistic coordination between miRNAs and BA metabolism in maintaining intestinal homeostasis. Among IBD-associated miRNAs, the miR-29 family (miR-29a/b/c) has been persistently identified as differentially expressed in the colonic mucosa from both UC and CD cohorts, with established roles in intestinal inflammation, immune tolerance, and anti-fibrosis (Lv et al., 2014; Nijhuis et al., 2014). Notably, the promoter regions of miR-29 family members contain predicted binding sites for key transcription factors regulating BA metabolism and receptor signaling, such as FXR, PXR, and VDR (Li et al., 2011; Panizo et al., 2017). These findings suggest a potential epigenetic link between miR-29a and BA metabolism or BA-responsive signaling. However, whether miR-29a modulates LCA-mediated intestinal homeostasis in IBD remains unexplored. Because miR-29a is encoded within the *Mir29a/Mir29b-1* locus, we primarily used a locus-deficient mouse model to examine alterations in gut microbial composition and BA metabolism during colitis, and combined this *in vivo* approach with miR-29a mimic and inhibitor experiments in Caco-2 cells. Here, our observations implicate miR-29a as a host factor associated with dextran sulfate sodium (DSS)-induced inflammatory responses and alterations in microbiota-associated BA metabolism, particularly the CDCA/LCA balance. Our study supports further investigation of miR-29a and LCA-targeted strategies in IBD.

## 2 Materials and methods

### 2.1 Mice

Animals were housed in a specific pathogen-free facility maintained at 22 ± 2 °C and 50 ± 5% humidity with a 12-h light/dark cycle. Mice received an AIN-93M diet (Ready Dietech Inc., Shenzhen, China) and autoclaved water ad libitum.

*Mir29a/Mir29b-1* locus-deficient C57BL/6J mice were generated with a CRISPR/Cas9 approach as described previously, by Shanghai Model Organisms Center Inc. (Shanghai, China) (Zhu et al., 2019). The approximately 600-bp deletion encompassed the complete Mir29a and Mir29b-1 sequences. Because the independent Mir29b-2/Mir29c cluster was not targeted, this model completely eliminates miR-29a while retaining a genomic source of mature miR-29b. Genotypes were confirmed by PCR amplification of tail DNA using primers 5’-AGGGGGCAGGGTCTCATTAGCA-3’ and 5’-CCCACCCCCTTCCCCTCAG-3’ to ensure homozygous or WT status (**Supplementary Figure 1**). All animal procedures were performed under the relevant animal protocol (no. gdpulacSPF2017118), which was approved by the Institutional Animal Care and Use Committee (IACUC) of Guangdong Pharmaceutical University (Guangzhou, China).

### 2.2 DSS-induced colitis model

Acute colitis was induced (Wirtz et al., 2017) in 10-week old male mice by exposing to 2% (w/v) DSS (MW 36–50 kDa; MP Biomedicals, Solon, OH, USA) in drinking water for 7 days. WT and knockout (KO) mice were randomized into WT control, KO control, WT DSS and KO DSS cohorts (n = 9 per group, except n = 8 in the KO-DSS group). The control groups were included to assess genotype-related baseline differences, whereas the DSS-treated groups allowed evaluation of DSS-induced colitis in each genotype and the effect of miR-29a/b1 deficiency under inflammatory conditions. Body weight and disease activity index (DAI) were recorded every other day; DAI was calculated from weight loss, rectal bleeding, and stool consistency, as described in **Supplementary Table 1** (Wirtz et al., 2017). Together with colon length, histopathological assessment, and colonic inflammatory gene expression, these measures were used to evaluate colitis induction and colonic injury. 7 days after exposure to DSS, mice were placed in an induction chamber and anesthetized with 2-3% isoflurane delivered by a precision vaporizer until deep anesthesia was achieved. Anesthesia was subsequently maintained with isoflurane via a nose cone during terminal blood collection. Cervical dislocation was then performed immediately, followed by collection of colon and fecal samples. **Figure 1A** illustrates the experimental schedule.

**Figure 1:**
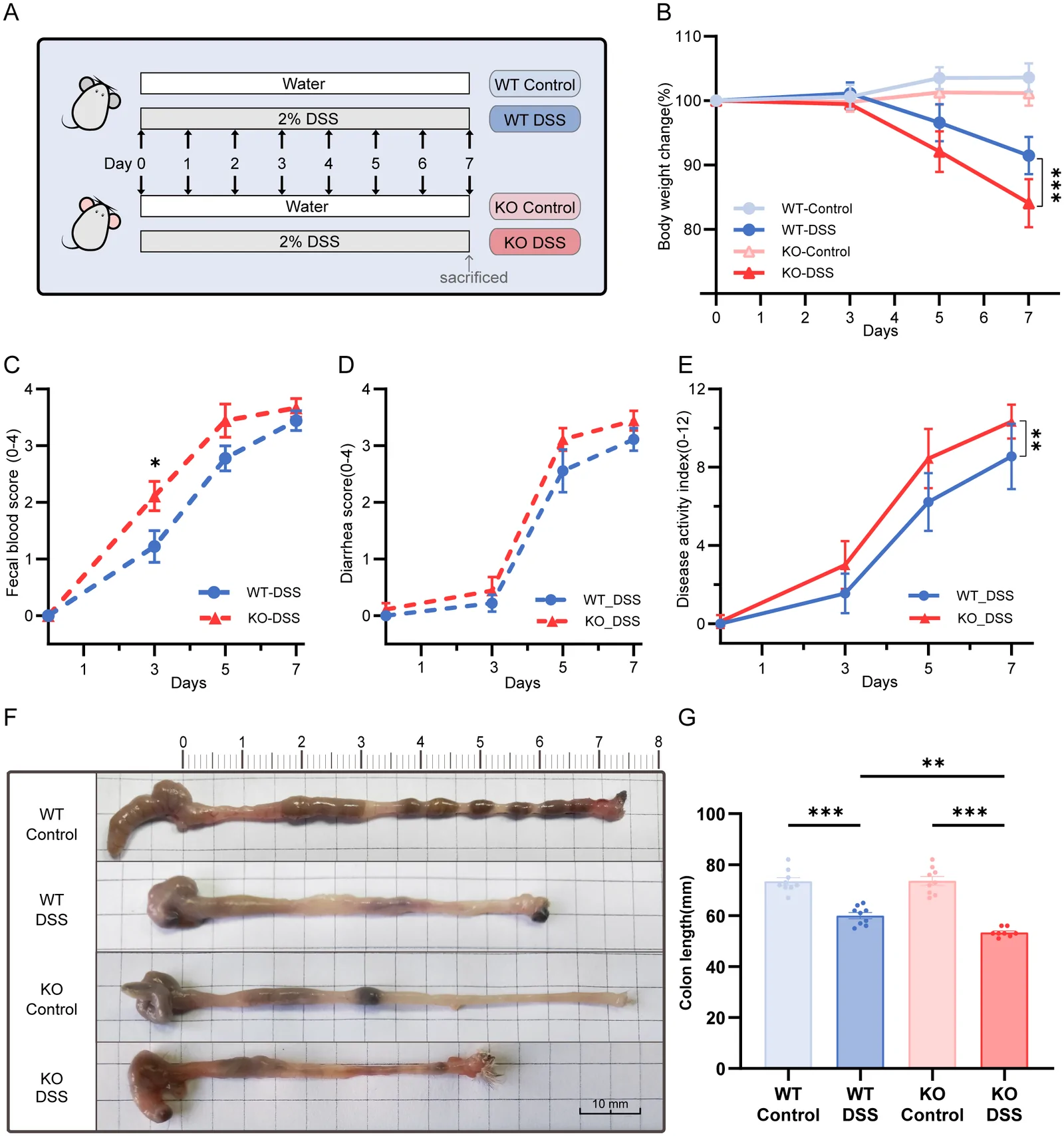
miR-29a/b1 deficiency aggravates the progression of DSS-induced colitis. **(A)** Illustrative outline of the DSS-induced colitis model. **(B)** Body weight changes during DSS administration. Values are presented as the percentage of the initial weight. **(C)** Rectal bleeding and **(D)** diarrhea score. **(E)** DAI measurements for experimental groups. Mice were sacrificed 7 days after DSS exposure. **(F)** Macroscopic colonic images (scale bar: 10 mm) and **(G)** quantification of colon lengths. Results are represented as mean ± SD (*n =* 9 per group, excluding *n =* 8 in the KO-DSS group). Statistical analysis: B-E, Mann-Whitney U test; G, one-way ANOVA with Tukey’s *post hoc* test. *p < 0.05, **p < 0.01, ***p < 0.001. WT, wild type; KO, knockout; DSS, dextran sulfate sodium; DAI, Disease activity index.

### 2.3 Histology

Colonic samples were collected, fixed in paraformaldehyde, and embedded in paraffin. 5 μm sections were stained with hematoxylin and eosin to perform histological examination. Colon injury assessment was performed by an experienced histologist blinded to mouse strain and treatment group. To evaluate the severity of histologic colitis, a previously detailed semiquantitative scoring system (Zhao et al., 2022) was utilized and is described in **Supplementary Table 2**.

### 2.4 Ultrastructural observation by TEM

Colon tissues were sliced into 1 mm^3^ pieces and promptly fixed in 2.5% glutaraldehyde (4 °C, 4 h), followed by post-fixation in 2% osmium tetroxide (0.1 M PBS, pH 7.4, 2 h). After dehydration through a graded ethanol series, tissues were embedded in Epon 812. Ultrathin sections of 75 nm were cut and examined using an electron microscope (HT7700, Hitachi, Japan). Microvilli length (μm) in differentiated colonocytes was manually quantified through morphometric analysis using ImageJ software. For each group, 6 mature colonocytes and a median of 67.75 (range: 28-90) microvilli were counted.

### 2.5 16S rRNA gene sequencing and bioinformatic analysis

Total genomic DNA was extracted from fecal samples using the SDS method following previous method (Zhao et al., 2022). The V3-V4 region of the bacterial 16S rRNA gene was amplified using barcoded primers 343F (5’-TACGGRAGGCAGCAG-3’) and 798R (5’-AGGGTATCTAATCCT-3’) (Nossa et al., 2010). Amplicon libraries were sequenced as single end reads on an Ion S5 XL platform by Novogene Technology Co., Ltd. (Beijing, China).

After quality filtering and chimera removal, clean reads were clustered into operational taxonomic units (OTUs) at 97% sequence similarity using UPARSE, and representative sequences were taxonomically annotated against the SILVA database using the mothur algorithm. The OTU abundance table was normalized to the minimum sequencing depth across samples. Alpha diversity and rarefaction curves were generated using QIIME, whereas beta diversity was evaluated using Bray-Curtis dissimilarities and visualized by principal coordinate analysis (PCoA). Microbial functional potential was inferred using PICRUSt2. Detailed amplification, library preparation, and sequence-processing procedures are provided in the **Supplementary Methods**. The sequencing data were deposited in the Genome Sequence Archive (GSA) under accession CRA040687 (CNCB-NGDC Members and Partners, 2025; Zhang et al., 2025).

### 2.6 Cell culture and reagents

Caco-2 cells sourced from Chinese Academy of Science Cell Bank, were kept in MEM with the addition of 20% FBS and 1% NEAA. DSS-containing medium was passed through a 0.22-μm filter before use. For DSS-induced colonic inflammation cell model (Gadaleta et al., 2011; Toutounji et al., 2020; Roselli et al., 2022), Caco-2 cells were incubated with or without 2% DSS for 48 h, either in the presence or absence of 50 μM LCA (HY-B0172; MedChemExpress, Monmouth Junction, NJ, USA). Transfection procedures were carried out when Caco-2 cells reached 70% confluence in 6-well plates. MiRNA-mimic negative control(NC), miRNA-inhibitor NC, miR-29a mimic (50nM) or miRNA-29a inhibitor (200 nM) (Ribobio Bio, Beijing, China) were applied for transfection by using Lipofectamine 3000 (Invitrogen, Carlsbad, CA, USA) as describe before (Zhu et al., 2019). Following overnight transfection, the culture medium was replaced, and the indicated DSS and/or LCA treatments were initiated. For co-treatment, DSS and LCA were added simultaneously.

For the cell viability assay, Caco-2 cells were seeded in 96-well plates(2 × 10³ cells/well) overnight, then treated with various concentrations of LCA (0, 25, 50, 100, and 200 μM) or DMSO. Cell viability was assessed after 24, 48, and 72 h of incubation using an MTS kit (Promega, Madison, WI, USA) whereby the optical density (OD) was recorded at 490 nm.

### 2.7 RT-qPCR for miRNA and mRNA

Total miRNA was isolated using the miRcute Kit (DP501; Tiangen, Beijing, China) and reverse-transcribed via the miRcute Plus First-Strand Kit (KR211; Tiangen). RT-qPCR amplification was subsequently performed using the miRcute Detection Kit (FP411-01; Tiangen).

Total RNA was extracted from Caco-2 cells and mouse colon tissues using TRIzol reagent and converted to cDNA via the PrimeScript RT Master Mix (RR036A, Takara, Kusatsu, Japan). Real-time PCR was then conducted with the SYBR Green PCR Master Mix (RR082A; Takara) on an ABI 7500 platform.

All RNA extracted from DSS-treated cells and mouse colon tissues was purified via lithium chloride precipitation (Viennois et al., 2018) prior to reverse transcription. Expression was normalized to U6 for miRNA, GAPDH for Caco-2 mRNA, and Gapdh and Actb for mouse colonic mRNA, and determined using the 2^−ΔΔCt^ method. Cell RT-qPCR experiments comprised three independent biological replicates, whereas mouse colonic analyses included 7–8 mice per group. Primer sequences are listed in **Supplementary Table 3**.

### 2.8 Determination of BAs

Fecal BA profiling was conducted using 6 mice in each of the WT-Control, WT-DSS, and KO-Control groups and 4 mice in the KO-DSS group, by Lu-Ming Biotech Co., Ltd. (Shanghai, China) using a UPLC-MS/MS platform. A mixed BA standard in 1:1 (v/v) methanol-water was used for calibration. Chromatographic separation was achieved on an ExionLC system (AB Sciex, Shanghai, China) using a C18 column with a mobile phase gradient of water and organic solvents (0.1% formic acid). Mass spectrometry was conducted using an AB Sciex Qtrap 6500+ system with electrospray ionization in dual ion modes. Multiple reaction monitoring was employed for BA detection, with data processed via Analyst and Sciex OS-MQ software. Final BA concentrations were normalized to dry fecal mass (per gram of dry feces). 41 BAs were determined and the details are listed in **Supplementary Table 4**. Quantified BAs were classified as primary or secondary as specified in **Supplementary Table 4**, and the proportion of each category was calculated relative to the total BA concentration included in the pool analysis. Total CDCA and total LCA were calculated as the sums of their unconjugated, glycine-conjugated, and taurine-conjugated forms. The LCA/CDCA and DCA/CA ratios were calculated from the corresponding dry-weight-normalized concentrations.

### 2.9 Statistical analysis

Results are expressed as mean ± SD, unless otherwise indicated. Normality and homogeneity of variance were assessed using the Shapiro-Wilk and Brown-Forsythe tests, respectively. For two-group comparisons, Student’s t-test was used for normally distributed data with homogeneous variances, Welch’s t-test for normally distributed data with unequal variances, and the Mann-Whitney U test for non-normally distributed data. For multiple group comparisons, one-way ANOVA with Tukey’s *post hoc* test was used for normally distributed data with homogeneous variances, Welch’s one-way ANOVA with Dunnett’s T3 *post hoc* test was used for normally distributed data with unequal variances, and the Kruskal-Wallis test with Dunn’s *post hoc* test was applied to non-normally distributed data. For microbiome analyses, differences in overall community composition based on Bray-Curtis dissimilarities were assessed using permutational multivariate analysis of variance (PERMANOVA), and homogeneity of multivariate dispersion was evaluated using PERMDISP. Genus-level differences were assessed using the Kruskal-Wallis test with Benjamini-Hochberg false-discovery rate correction, followed by Holm-adjusted Dunn’s *post hoc* tests. Associations between genus-level relative abundances and colitis-related indices were evaluated using Spearman’s rank correlation with Benjamini-Hochberg correction. To examine the relationship between fecal BA profiles and colitis-related indices, Spearman’s rank correlation was conducted. General statistical analyses and graphical representation were performed using GraphPad Prism 8.4.3. Microbiome-related data processing and statistical analyses were conducted in Python 3 using the pandas, NumPy, SciPy, scikit-posthocs and statsmodels packages. Additional correlation and clustering analyses, as well as data visualization, were performed in R 3.6.3 using the stats, ggplot2 and pheatmap packages. All tests were two-sided, and *p* values or adjusted *q* values <0.05 were considered statistically significant.

## 3 Results

### 3.1 miR-29a/b1 deficiency exacerbates the severity of DSS-induced colitis in mice

For analysis of the potential function of miR-29a/b1 in intestinal inflammation, we subjected WT and miR-29a/b1 KO mice to an established experimental colitis model induced by 2% DSS for 7 days (**Figure 1A**). Under water-treated conditions, no significant differences in body weight change or colon length were observed between WT-Control and KO-Control mice (**Figures 1B, F, G**). DSS administration induced significant disease manifestations characteristic of colitis, which were remarkably aggravated in the absence of miR-29a/b1. DSS-treated mice showed progressive body-weight loss, with a significantly greater reduction in KO-DSS than in WT-DSS mice (*p <* 0.001, **Figure 1B**). Furthermore, miR-29a/b1 deficiency led to a deterioration of clinical manifestations associated with colitis, as evidenced by an earlier onset of severe rectal bleeding (*p <* 0.05 on day 3, **Figure 1C**), increased diarrhea scores (**Figure 1D**), and a significantly higher overall Disease Activity Index (DAI) by the end of the DSS treatment (**Figure 1E**).

Macroscopic evaluation of the murine colons further corroborated the *in vivo* phenotypic observations. DSS treatment significantly shortened colon lengths in both genotypes compared to their respective water-treated controls (**Figures 1F, G**); colon length was also significantly shorter in KO-DSS than in WT-DSS mice (**Figures 1F, G**). Collectively, these physiological and macroscopic alterations indicate that loss of miR-29a/b1 enhances vulnerability to intestinal inflammation and aggravates the progression of DSS-induced colitis.

### 3.2 miR-29a/b1 deficiency aggravates DSS-induced colonic injury and inflammatory responses

To further elucidate the role of miR-29a/b1 in maintaining colonic structural integrity, histological profiling of the distal colon was conducted via H&E staining. While the colonic tissues of water-treated WT and KO mice exhibited normal crypt architecture and intact epithelium, DSS exposure provoked severe pathological manifestations, including epithelial layer destruction, crypt ablation, and dense inflammatory cell infiltration (**Figure 2A**). Especially, these pathological changes were amplified in the KO-DSS group when compared against their WT-DSS counterparts, a distinction quantitatively validated by elevated histological scores (**Figure 2B**). These findings support a role for miR-29a/b1 in limiting DSS-induced colonic mucosal injury. Ultrastructural analysis via transmission electron microscopy (TEM) was conducted to evaluate epithelial barrier integrity at a subcellular resolution. These ultrastructural assessments indicated that DSS administration disrupted the apical junctional complexes (tight junctions, adherens junctions, desmosomes) and resulted in shortened, disorganized microvilli in WT mice compared to their controls (**Figures 2C, D**). In KO mice, DSS exposure led to severe ultrastructural deterioration, characterized by severely fragmented microvilli and morphologically disrupted cell junctions (**Figure 2C**).

**Figure 2:**
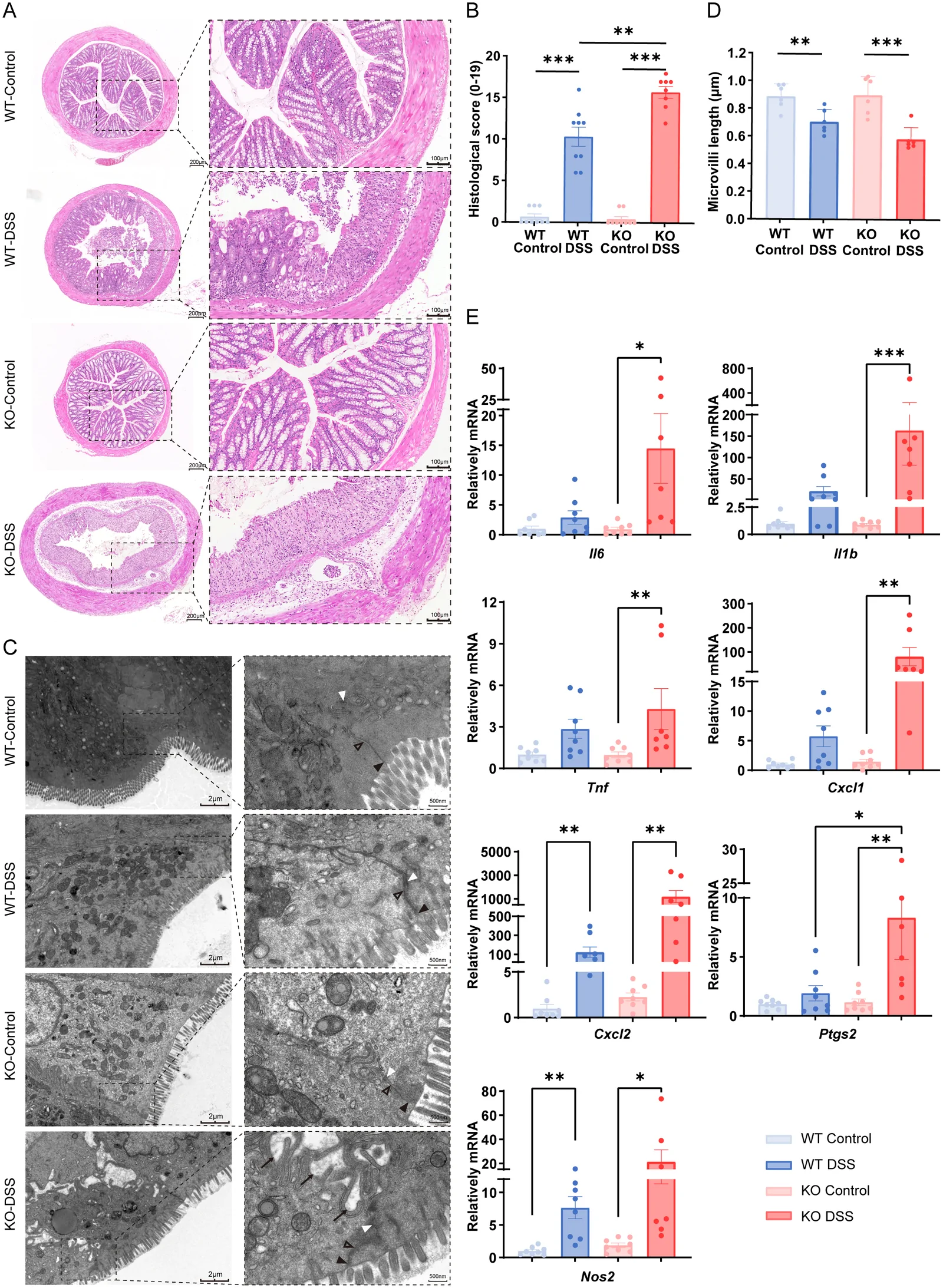
miR-29a/b1 deficiency exacerbates DSS-induced colonic injury and inflammatory responses. **(A)** Colon tissue micrographs with H&E staining. Scale bars: 200 μm (left), 100 μm (right). **(B)** Histopathology scores. *n =* 9 per group, excluding *n =* 8 in DSS-treated KO group. **(C)** Representative TEM images of intestinal epithelium ultrastructure. Scale bars: 2 μm (left), 500 nm (right). Black triangles: TJs; hollow triangles: AJs; white triangles: Des. Arrows indicate disrupted junctional structures. **(D)** Quantification of microvilli length. *n =* 6 per group. **(E)** Relative mRNA expression of *Il6*, *Il1b*, *Tnf*, *Cxcl1*, *Cxcl2*, *Ptgs2* and *Nos2* in colon tissues, normalized to *Gapdh* and *Actb* (n = 7–8 mice per group). Data are presented as mean ± SD. Statistical analysis: B and D, Welch’s one-way ANOVA with Dunnett’s T3 *post hoc* test; E, Kruskal-Wallis test with Dunn’s *post hoc* test. *p < 0.05, **p < 0.01, ***p < 0.001. H&E, hematoxylin and eosin; TEM, transmission electron microscopy; TJ, tight junction; AJ, adherens junction; Des, desmosome; WT, wild type; KO, knockout; DSS, dextran sulfate sodium.

To further assess colonic inflammatory responses, the expression of inflammatory genes was examined in colon tissues. In KO mice, DSS treatment significantly increased the expression of *Il6*, *Il1b*, *Tnf*, *Cxcl1*, *Cxcl2*, *Ptgs2*, and *Nos2* compared with KO-Control mice (**Figure 2E**). In WT mice, DSS treatment significantly increased *Cxcl2* and *Nos2* expression compared with WT-Control mice. Notably, *Ptgs2* expression was significantly higher in KO-DSS mice than in WT-DSS mice. The remaining inflammatory transcripts showed higher mean expression in KO-DSS mice, although the between-group differences were not statistically significant (**Figure 2E**). Together, these histological, ultrastructural, and gene-expression findings indicate that miR-29a/b1 deficiency is associated with aggravated colonic injury and inflammatory responses during DSS-induced colitis.

### 3.3 miR-29a attenuates DSS-induced inflammation in Caco-2 cells

To directly explore the regulatory function of miR-29a on intestinal epithelial inflammation *in vitro*, human Caco-2 cells were utilized. The transfection efficiency was monitored with a Cy3-labeled negative control (Cy3-mimic NC and Cy3-inhibitor NC), demonstrating high transfection efficiency and expected alterations in miR-29a expression following mimic or inhibitor treatment (**Figures 3A, B**). Concurrently, to simulate epithelial damage and cellular stress *in vitro*, a 48 h administration of 2% DSS was applied to the Caco-2 cell line (**Figure 3C**).

**Figure 3:**
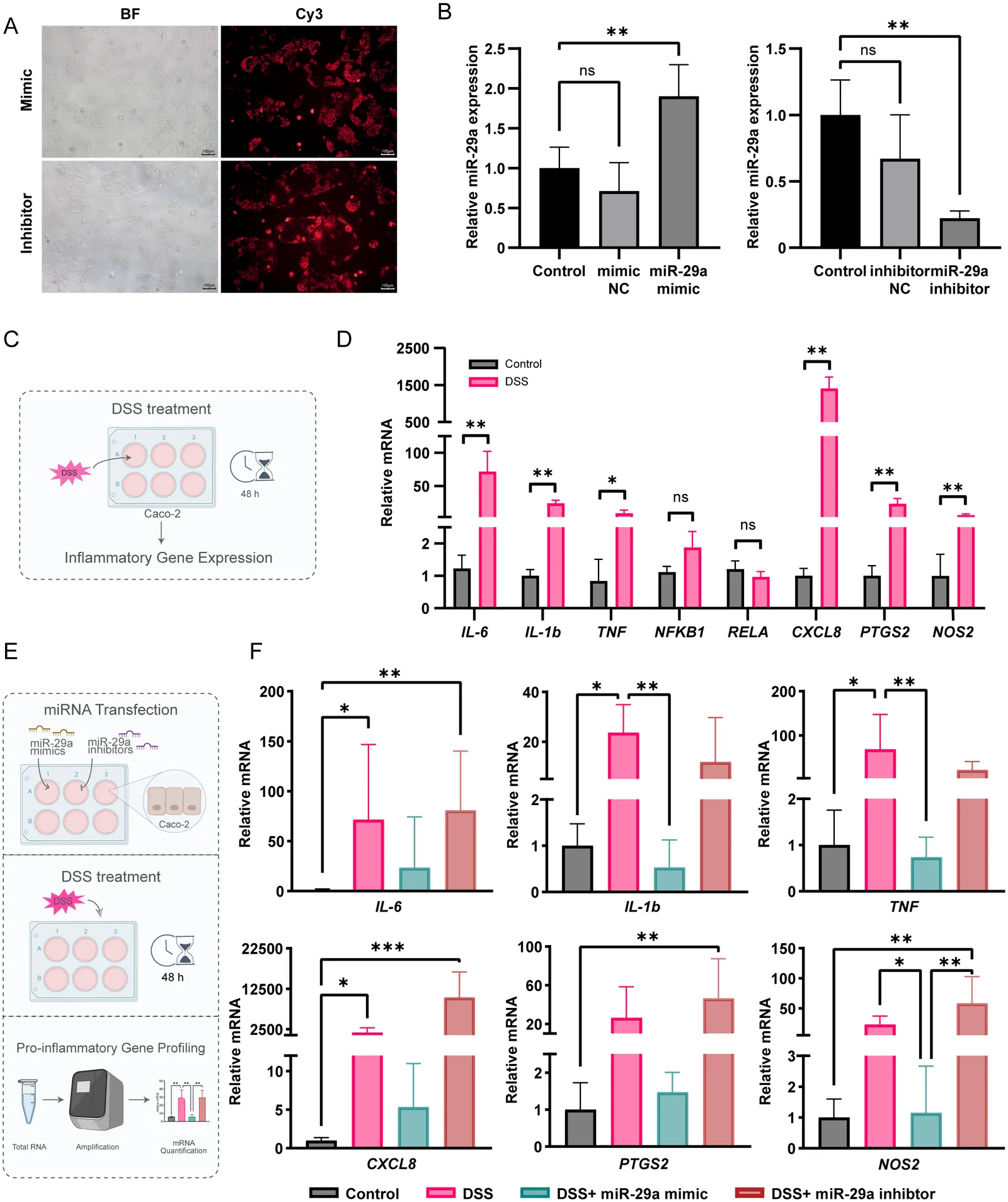
miR-29a alleviates DSS-induced inflammatory responses in intestinal epithelial cells *in vitro*. **(A)** Caco-2 cells were transfected with Cy3-labeled mimic or inhibitor negative controls to confirm transfection efficiency. **(B)** RT-qPCR assessment of Caco-2 cells transfected with specific oligonucleotides (miR-29a mimic, mimic NC, miR-29a inhibitor, inhibitor NC). **(C)** Schematic diagram of cell experiments. Caco-2 cells were exposed to 2% DSS or vehicle control for 48 h, followed by examination of inflammation markers by RT-qPCR. **(D)** Levels of mRNA expression for inflammation- associated genes *IL6*, *IL1B*, *TNF*, *NFKB1*, *RELA*, *CXCL8*, *PTGS2*, and *NOS2* in Caco-2 cells. **(E)** Experimental workflow for miR-29a modulation. Following transfection with either inhibitors or mimics of miR-29a, Caco-2 cells were incubated with or without 2% DSS for 48 h before detecting inflammatory markers by RT-qPCR. **(F)** Levels of mRNA expression for pro-inflammatory genes in Caco-2 cells following exposure to baseline conditions, DSS, or DSS in combination with either miR-29a mimics or inhibitors. *U6* and *GAPDH* were used to normalize miRNA and mRNA expression, respectively. Data are presented as mean ± SD from 3 independent biological experiments. Statistical analysis: B, Welch’s one-way ANOVA with Dunnett’s T3 *post hoc* test; D, Welch’s t-test for *IL1B*, *RELA*, *CXCL8*, and Mann-Whitney U test for *IL6*, *TNF*, *NFKB1*, *PTGS2*, *NOS2*; F, Kruskal-Wallis test with Dunn’s *post hoc* test. *p < 0.05, **p < 0.01, ***p < 0.001. ns, not significant; BF, bright field; NC, negative control; DSS, dextran sulfate sodium.

To characterize the DSS-induced epithelial inflammatory response, we examined gene expression of representative inflammatory cytokines, chemokines and effector enzymes. RT-qPCR analysis verified that exposure to DSS provoked a marked upregulation of mRNA expression levels for multiple pro-inflammatory genes compared to the untreated control (**Figure 3D**), comprising the cytokines interleukin 6 (*IL6*), interleukin 1 beta (*IL1B*), tumor necrosis factor (*TNF*), and interleukin 8 (*CXCL8*), as well as inflammatory enzymes, including prostaglandin-endoperoxide synthase 2 (*PTGS2*) and nitric oxide synthase 2 (*NOS2*). Subsequently, the influence of modulating cellular miR-29a levels on the DSS-induced inflammatory response was investigated (**Figure 3E**). The findings revealed that miR-29a overexpression significantly attenuated the DSS-induced upregulation of *IL1B*, *TNF*, and *NOS2* (**Figure 3F**). *IL6*, *CXCL8*, and *PTGS2* showed lower mean expression in the DSS + miR-29a mimic group than in the DSS-only group, although the differences were not statistically significant. Conversely, *IL6*, *CXCL8*, *PTGS2*, and *NOS2* showed higher mean expression in the DSS + miR-29a inhibitor group than in the DSS-only group, but these differences did not reach statistical significance. *NOS2* expression was significantly higher in the inhibitor group than in the mimic group (**Figure 3F**). Collectively, these *in vitro* data support an inhibitory role for miR-29a in DSS-induced inflammatory responses in intestinal epithelial cells.

### 3.4 Deletion of miR-29a/b1 exacerbates DSS-induced gut microbiota dysbiosis

Recognizing the crucial role of the gut microbiome in sustaining mucosal equilibrium, fecal 16S rRNA amplicon sequencing was utilized to investigate how miR-29a/b1 deficiency alters bacterial community structures. After quality filtering, 80,013-80,314 clean reads were retained per sample. Good’s coverage was 0.999 for all samples, and the rarefaction curves approached a plateau, indicating adequate sequencing depth (**Supplementary Table 5** and **Supplementary Figure 2A**). Investigation of operational taxonomic units (OTUs) disclosed 317 shared OTUs among the 4 experimental groups, alongside distinct unique OTUs in each group (**Figure 4A**). Alpha diversity analysis showed that DSS administration reduced microbial richness and diversity, with the lowest values generally observed in the KO-DSS group. KO-DSS mice showed significantly lower Observed species (**Figure 4B**), Chao1, ACE, and PD_whole_tree indices (**Supplementary Figure 2B**) in the indicated group comparisons. The Simpson index was also significantly reduced in KO-DSS mice, whereas the mean Shannon index was lower than those in the other groups but did not differ significantly (**Figure 4B**). Bray-Curtis based principal coordinate analysis (PCoA) showed group-related differences in microbial community structure, with a significant overall group effect by exact PERMANOVA (pseudo-F = 4.172, R² = 0.610, exact p = 0.000065; **Figure 4C**; **Supplementary Table 6**). Multivariate dispersion also differed significantly among groups (PERMDISP F = 23.070, exact p = 0.0038), with greater within-group dispersion in the KO-DSS group. Thus, the overall beta-diversity difference may reflect both differences in community composition and unequal within-group variability.

**Figure 4:**
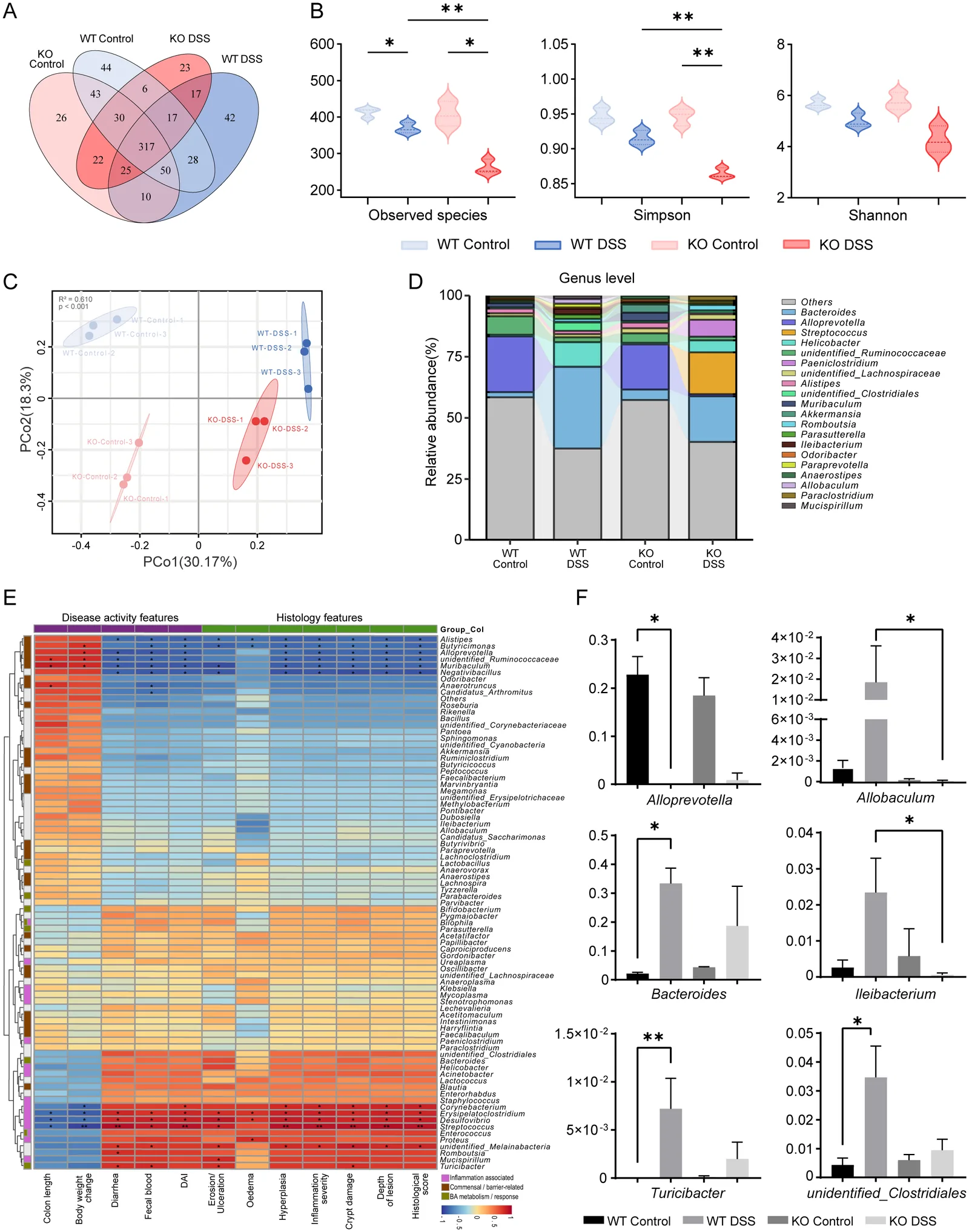
Alterations in fecal microbiota between WT and KO mice under baseline and inflammatory conditions. **(A)** Venn diagram illustrating the overlapping and unique OTUs among the four groups. **(B)** Observed species, Simpson and Shannon index represent the alpha diversity indices. **(C)** Principal coordinate analysis based on Bray-Curtis dissimilarities. **(D)** Relative abundances of the 20 most abundant genera. **(E)** Spearman correlation heatmap showing associations between genus-level relative abundances and colitis-related indices. Colors indicate Spearman’s rho, and asterisks indicate Benjamini-Hochberg adjusted q values. **(F)** Relative abundances of *Alloprevotella*, *Allobaculum*, *Bacteroides*, *Ileibacterium*, *Turicibacter*, and *unidentified_Clostridiales*, which met the Holm-adjusted Dunn’s *post hoc* threshold in the pairwise comparisons. Data are represented as mean ± SD. *n =* 3. Statistical analysis: B, Kruskal-Wallis test with Dunn’s *post hoc* test; C, exact PERMANOVA. B: *p < 0.05, **p < 0.01; E: *q < 0.05, **q < 0.01; F: *adjusted *p* < 0.05, **adjusted *p* < 0.01. WT, wild type; KO, knockout; DSS, dextran sulfate sodium; OTU, operational taxonomic unit; PCoA, principal coordinate analysis; PERMANOVA, permutational multivariate analysis of variance; DAI, diseaseactivity index.

To characterize overarching structural changes, the gut microbiota taxonomic composition was initially evaluated at the phylum level. Administering DSS led to modifications in the relative abundances of the dominant phyla, with Firmicutes and Bacteroidetes being primarily affected (**Supplementary Figure 2C**). To more precisely identify the specific bacterial taxa driving these community shifts, a granular taxonomic evaluation was subsequently conducted to characterize these populations down to the genus classification. The overall composition of the 20 most abundant microbial genera was altered across the groups (**Figure 4D**). Furthermore, Spearman correlation analysis revealed distinct associations between genus-level relative abundances and colitis-related clinical and histological indices; of the 960 correlations tested, 119 remained significant after Benjamini-Hochberg correction, involving 17 genera and all 12 phenotypic indices (**Figure 4E**).

At the genus level, none of the tested taxa remained significant after Benjamini-Hochberg correction of the global Kruskal-Wallis tests (**Supplementary Table 7**). **Figure 4F** presents all taxa meeting the within-taxon Dunn-Holm threshold in the three prespecified pairwise comparisons. Relative to WT-Control mice, WT-DSS mice exhibited a lower relative abundance of *Alloprevotella* and higher relative abundances of *Bacteroides*, *Turicibacter*, and *unidentified_Clostridiales*. In addition, *Allobaculum* and *Ileibacterium* were less abundant in KO-DSS mice than in WT-DSS mice. Although none of the corresponding global genus-level tests remained statistically significant after FDR correction, the taxa identified in the prespecified pairwise comparisons included genera with potential metabolic relevance. In particular, *Bacteroides*, a predominant genus associated with microbial BA metabolism, was enriched in WT-DSS mice relative to WT-Control mice and showed lower mean abundance in KO-DSS mice than in WT-DSS mice, although the latter difference was not statistically significant. Together with the broader community-level alterations, these findings prompted us to examine whether miR-29a/b1 deficiency was accompanied by changes in predicted microbial BA-metabolic potential and fecal BA profiles.

### 3.5 Alteration of fecal BA metabolism and decreased proportion of secondary BAs in miR-29a/b1-deficient colitis mice

Given the indispensable role of the intestinal microbiome in mediating BA biotransformation, we investigated whether the observed microbial dysbiosis in miR-29a/b1-deficient mice affected fecal BA profiles. PICRUSt2-based inference of microbial functional potential showed that the predicted relative abundance of the bile salt hydrolase gene (*bsh*) was significantly lower in KO-DSS mice than in WT-DSS mice. The predicted relative abundance of the 7α-hydroxysteroid dehydrogenase gene (*hdhA*) was also lower on average, although the difference was not statistically significant (**Figure 5A**). Consistent with these functional predictions, targeted metabolomics further revealed differences in the overall fecal BA profiles among the four groups (**Figure 5B**). Specifically, when BA species were classified as primary or secondary and expressed as proportions of the total fecal BA pool, KO-DSS mice showed a significantly higher primary BA proportion and a significantly lower secondary BA proportion than WT-DSS mice (**Figure 5D**). These data indicate that the bacteria-mediated biotransformation from primary into secondary BAs is compromised in the absence of miR-29a/b1.

**Figure 5:**
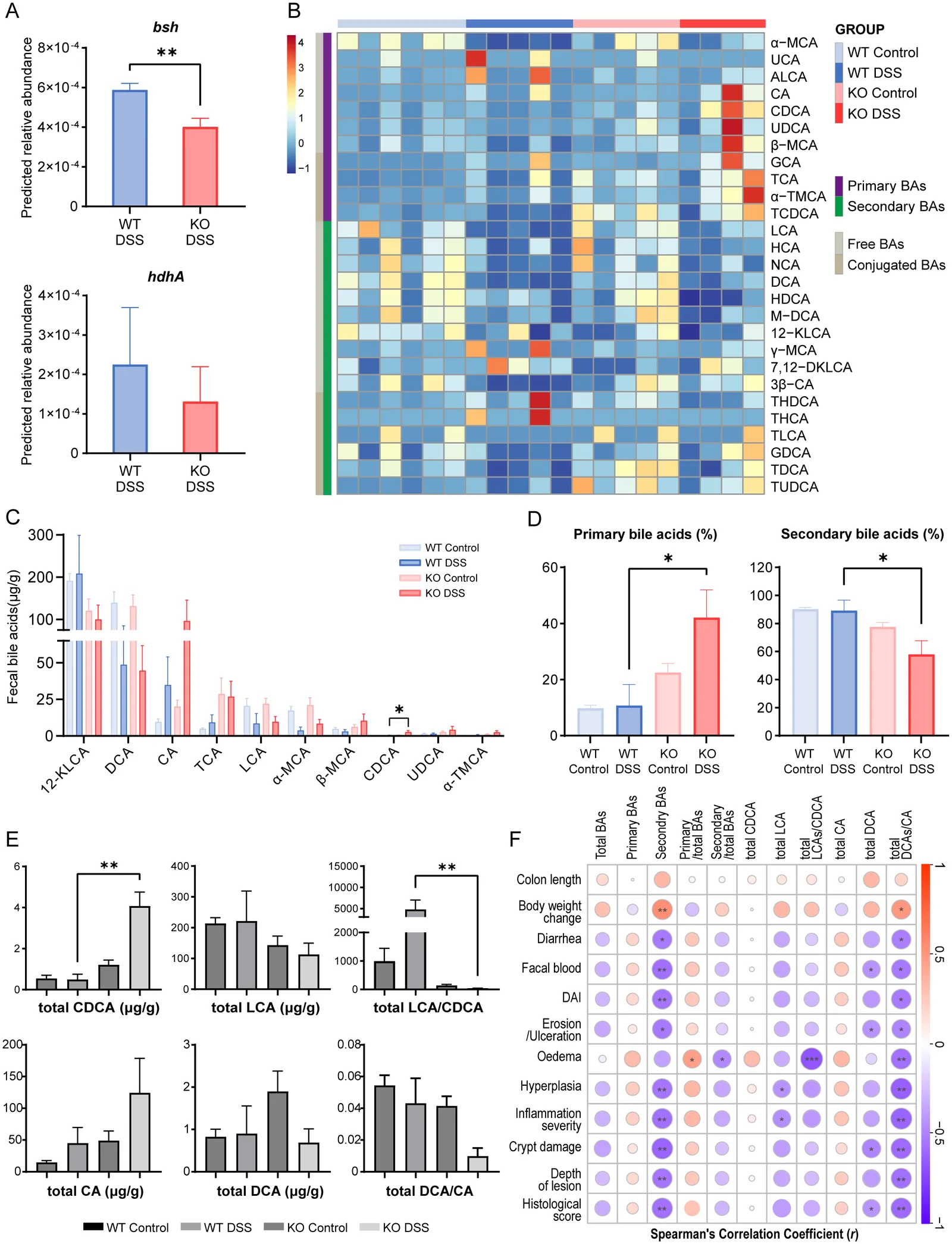
Dysregulation of BA metabolism in colitis mice. **(A)** PICRUSt2-predicted relative abundances of gut microbial *bsh* and *hdhA* genes in WT-DSS and KO-DSS groups. Differences between groups were analyzed using an unpaired Mann-Whitney U test. **(B)** Descriptive heatmap of fecal BA profiles. **(C)** Absolute concentrations of dominant fecal BAs (>0.2% of the total pool). β-MCA, CDCA, and α-TMCA were analyzed using ordinary one-way ANOVA with Tukey’s *post hoc* test; 12-KLCA, DCA, CA, TCA, LCA, α-MCA, and UDCA were analyzed using the Kruskal-Wallis test with Dunn’s *post hoc* test. **(D)** The relative proportions of primary and secondary BAs within the total fecal BA pool. Data were analyzed using the Kruskal-Wallis test with Dunn’s *post hoc* test. **(E)** Dry-weight-normalized concentrations of total CDCA, total LCA, total CA, and total DCA, and the corresponding LCA/CDCA and DCA/CA ratios. Total CDCA and the LCA/CDCA and DCA/CA ratios were analyzed using Welch’s one-way ANOVA with Dunnett’s T3 *post hoc* test; total LCA, total CA, and total DCA were analyzed using the Kruskal-Wallis test with Dunn’s *post hoc* test. **(F)** Spearman’s correlation between fecal BA measures and colitis-related indices. Values are expressed as mean ± SD. Sample sizes were n = 3 for A; and *n =* 6, excluding *n =* 4 for KO-DSS group in B-F. Brackets in A and C-E indicate the corresponding group comparisons; asterisks in F indicate Spearman correlation *p* values. *p < 0.05, **p < 0.01, ***p < 0.001. BA, bile acid; CDCA, chenodeoxycholic acid; LCA, lithocholic acid; CA, cholic acid; DCA, deoxycholic acid; bsh, bile salt hydrolase; hdhA, 7α-hydroxysteroid dehydrogenase; WT, wild type; KO, knockout; DSS, dextran sulfate sodium; DAI, disease activity index.

To assess pathway-specific product-to-precursor balance alongside absolute BA abundance, we next examined the dry weight normalized total CDCA, LCA, CA, and DCA concentrations and their corresponding ratios (**Figures 5C, E**). Total CDCA was significantly higher in KO-DSS mice than in WT-DSS mice, whereas total LCA did not differ significantly between the two DSS-treated groups. The total LCA/CDCA ratio was significantly lower in KO-DSS mice. In contrast, total CA, total DCA, and the total DCA/CA ratio did not differ significantly between WT-DSS and KO-DSS mice (**Figure 5E**). Spearman correlation analysis further showed that several secondary BA-related measures were associated with colitis phenotypes (**Figure 5F**). Secondary BA abundance and the total DCA/CA ratio were positively associated with body weight change and inversely associated with multiple clinical and histological severity indices. Within the CDCA-LCA pathway, total LCA was inversely associated with hyperplasia and inflammation severity, whereas the total LCA/CDCA ratio was inversely associated with oedema. Total CDCA showed no statistically significant associations with the assessed indices. Total DCA was also inversely associated with fecal blood, erosion/ulceration, crypt damage, and the overall histological score. Collectively, among the two major secondary BA pathways examined, CDCA-LCA showed significant between-group differences in both precursor abundance and the product-to-precursor ratio. Together with the inverse associations between total LCA and histological inflammatory indices, these findings identify an altered CDCA-LCA balance as a prominent BA-related feature of KO-DSS mice.

### 3.6 LCA mitigates DSS-induced epithelial inflammation while its efficacy is partially counteracted by miR-29a inhibition

Based on the altered CDCA-LCA balance and the inverse associations between LCA and inflammatory indices observed *in vivo*, we next investigated the effects of LCA on intestinal epithelial cells, with DCA included as another major secondary BA comparator. Initially, MTS assays established 50 µM (48 h) as the non-toxic concentration for subsequent Caco-2 cell experiments (**Figures 6A, B** and **Supplementary Figures 3A, B**). LCA treatment upregulated miR-29a expression in Caco-2 cells (**Figure 6C**), whereas DCA did not significantly alter miR-29a expression (**Figure 6C** and **Supplementary Figure 3C**).

**Figure 6:**
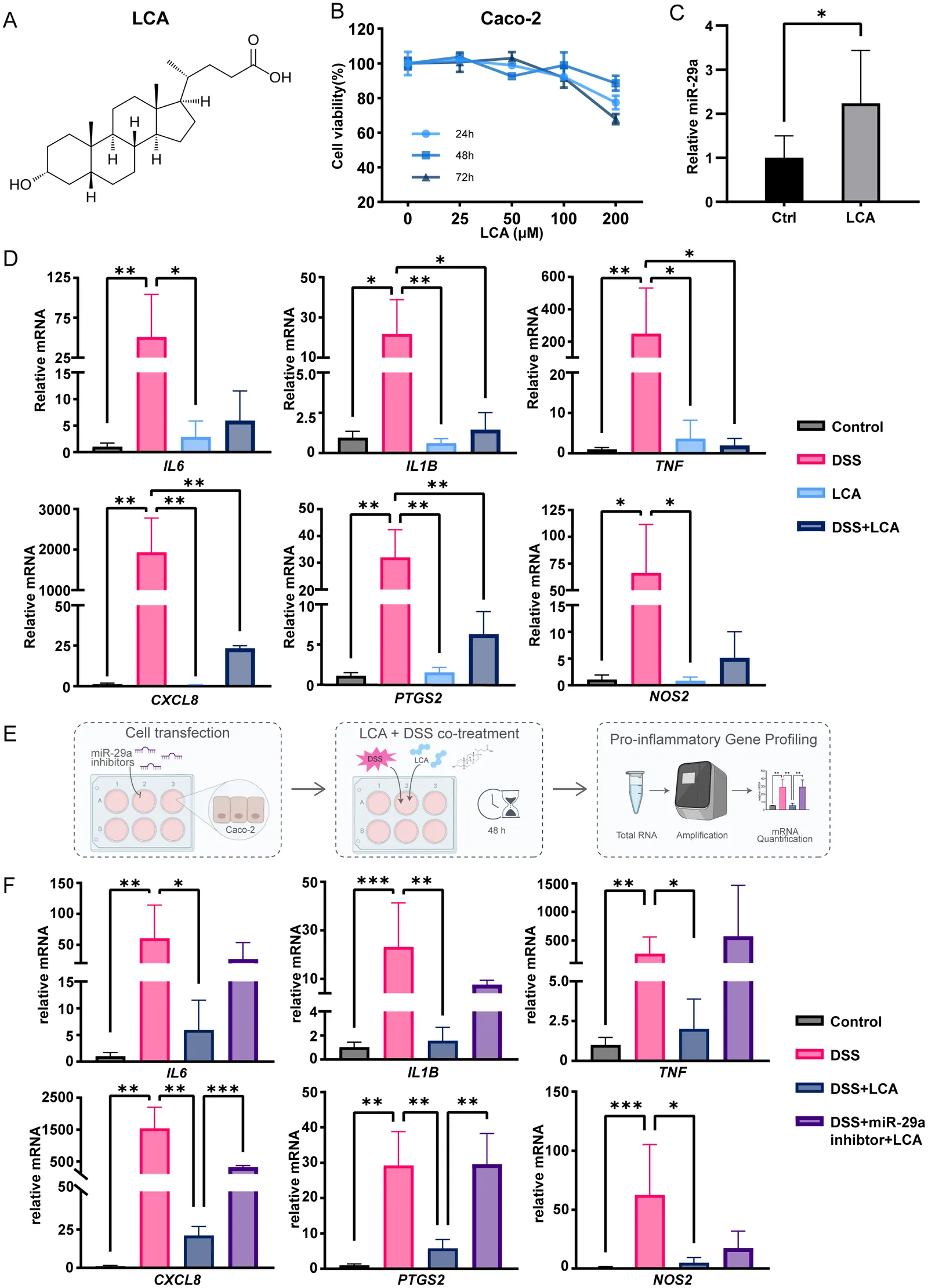
MiR-29a inhibition influences the anti-inflammatory activity of LCA in Caco-2 cells. **(A)** Chemical structure of LCA. **(B)** MTS assays were performed to determine Caco-2 cell viability after treatment with escalating concentrations of LCA (0, 25, 50, 100 and 200 μM) for the indicated durations (24, 48 and 72 h). **(C)** Relative miR-29a expression in LCA-treated Caco-2 cells. **(D)** RT-qPCR analysis of pro-inflammatory gene (*IL6*, *IL1B*, *TNF*, *CXCL8*, *PTGS2*, and *NOS2*) expression in Caco-2 cells following a 48-h incubation with 2% DSS and 50 μM LCA. **(E)** Schematic diagram illustrating the experimental design for miR-29a inhibition. Caco-2 cells treated with miR-29a inhibitors were incubated with 50 μM LCA and 2% DSS for 48 h before RT-qPCR analysis. **(F)** Relative expression of pro-inflammatory mRNAs (*IL6*, *IL1B*, *TNF*, *CXCL8*, *PTGS2*, and *NOS2*) in Caco-2 cells. Cells underwent Control, DSS, DSS + LCA, or DSS + miR-29a inhibitor + LCA treatments. Expression was normalized to *U6* (miRNA) or *GAPDH* (mRNA). Data are presented as mean ± SD from 3 independent biological experiments. Statistical analysis: C, Student’s t-test; D and F, Kruskal-Wallis test with Dunn’s *post hoc* test for *IL6*, *IL1B*, *TNF*, and Welch’s one-way ANOVA with Dunnett’s T3 *post hoc* test for *CXCL8*, *PTGS2*, *NOS2*. *p < 0.05, **p < 0.01, ***p < 0.001. LCA, lithocholic acid; DSS, dextran sulfate sodium; RT-qPCR, reverse transcription quantitative PCR.

Thereafter, Caco-2 cells underwent a 2% DSS challenge either with or without 50 µM LCA. RT-qPCR analysis showed that LCA treatment significantly attenuated the DSS-induced upregulation of the pro-inflammatory genes *IL1B*, *TNF*, *CXCL8*, and *PTGS2*, alongside a similar downward trend for *IL6* and *NOS2* (**Figure 6D**).

Furthermore, a miR-29a inhibitor was introduced into the DSS and LCA co-treatment system (**Figure 6E**). The suppression of endogenous miR-29a partially reversed the inhibitory effect of LCA on these pro-inflammatory genes. Specifically, compared to the DSS + LCA group, the miR-29a inhibitor broadly elevated these pro-inflammatory markers, with the restoration of *CXCL8* and *PTGS2* reaching statistical significance (**Figure 6F**). Collectively, these *in vitro* findings suggest that the anti-inflammatory ability of LCA on intestinal epithelial cells is functionally dependent on miR-29a.

## 4 Discussion

MiRNAs are crucial post-transcriptional regulators in the pathogenesis of IBD (Jung et al., 2021). Among them, the miR-29 family, particularly miR-29a, has attracted considerable interest because of its multifaceted roles in modulating intestinal fibrosis and epithelial homeostasis (Nijhuis et al., 2014; Zhou et al., 2015). Within the intestinal mucosa, miR-29a is prominently expressed in colonic epithelial cells, serving as a vital responder to cellular stress (Mo and Cao, 2023). Previous studies demonstrated significant upregulation of miR-29a in both UC patients and DSS-induced colitis mouse models (Lin et al., 2025). This increase may represent a compensatory response that helps limit severe colitis and preserve epithelial barrier integrity. Recent experimental *in vivo* studies have shown that targeted delivery of miR-29a, using carriers such as engineered nanoparticles or carbonate apatite complexes, exerts dual therapeutic effects against experimental colitis and intestinal fibrosis (Fukata et al., 2018; Niu et al., 2025). Consequently, restoring miR-29a levels has been proposed as a potential strategy for modulating intestinal inflammation in IBD. Under control conditions, WT-Control and KO-Control mice showed no significant differences in body-weight change or colon length, and both exhibited normal colonic histology. DSS exposure induced progressive weight loss, increased disease activity, colon shortening, and characteristic epithelial and crypt injury in both genotypes, consistent with established features of acute DSS-induced colitis (Wirtz et al., 2017; Zhao et al., 2022). These DSS-induced changes were more pronounced in KO-DSS mice, indicating that deletion of the *Mir29a/Mir29b-1* locus aggravated disease severity, colonic mucosal injury, and inflammatory responses. Although both precursor genes are deleted, the intact *Mir29b-2/Mir29c* locus can still generate mature miR-29b, leaving miR-29a as the only mature miR-29 family member completely eliminated in this model. Consistently, modulation of miR-29a expression *in vitro* further supported its inhibitory role in DSS-induced epithelial inflammatory responses.

While previous mechanistic investigations into the protective role of miR-29a have predominantly focused on host mucosal immunity, such as T cell regulation, or direct stromal fibrogenesis (Czopik et al., 2024; Lin et al., 2025), its relationship with the broader intestinal microenvironment, particularly microbial composition and microbiota-derived metabolites remains largely unexplored. Within our experimental model, miR-29a/b1 deficiency was associated with reduced microbial diversity and broader changes in community structure during DSS-induced colitis, similar to the reduced diversity and broader microbial perturbations reported during active IBD (Lloyd-Price et al., 2019). The genus-level correlations with clinical and histological indices also showed that these microbial changes tracked with disease severity, in line with previous evidence linking gut dysbiosis with inflammatory and immune responses (Kumar et al., 2025). Notably, the taxa highlighted in **Figure 4F** included *Bacteroides*, members of which are known to encode bile salt hydrolases, and *Turicibacter*, whose strains can directly modify host BA profiles (Lloyd-Price et al., 2019; Lynch et al., 2023). More importantly, these community-level changes were accompanied by predicted alterations in microbial BA-related functions (Lavelle and Sokol, 2020), consistent with recent evidence that microbiota-derived metabolite environments can influence epithelial inflammatory signaling under dysbiotic conditions (Dhiman et al., 2026). PICRUSt2 indicated a lower predicted abundance of *bsh* and a downward trend in *hdhA*, which are involved in BA deconjugation and structural conversion. In aggregate, these findings suggest that miR-29a/b1 deficiency is associated with a broader phenotype dysbiosis involving changes in microbial community structure and BA-metabolic potential during colitis.

Host metabolic homeostasis is profoundly shaped by the gut microbiota, in which BA biotransformation serves as a critical link (Lavelle and Sokol, 2020). Clinically, an abnormal accumulation of primary BAs and a concomitant depletion of secondary BAs represent one of the classical hallmarks of the IBD metabolome (Sinha et al., 2020), a metabolic imbalance that fundamentally deteriorates the intestinal micro-environment. Specifically, elevated levels of certain primary BAs can exert detergent-like effects on epithelial lipid bilayers and thereby compromise barrier integrity, whereas reduced availability of microbiota-derived secondary BAs may limit immunomodulatory signaling within the mucosa (Camilleri, 2020; Lavelle and Sokol, 2020).

Among these secondary BAs, LCA has emerged as a focal point of debate due to its context-dependent biological effects. On the one hand, certain studies indicate that highly hydrophobic LCA may exhibit potential cytotoxicity or pro-tumorigenic properties at supraphysiological concentrations (Lavelle and Sokol, 2020; Sinha et al., 2020; Wei et al., 2020). On the other hand, emerging evidences underscore its potent protective and anti-inflammatory functions in the colon at physiological levels (Ward et al., 2017; Fiorucci et al., 2021), which consistent with the anti-inflammatory effects observed in our study. We therefore examined the BA metabolic pattern in our model. Targeted metabolomic profiling revealed marked alterations in fecal BA composition associated with miR-29a/b1 deficiency. These alterations included a reduced proportion of secondary BAs in the fecal BA pool, accompanied by a significant increase in total CDCA and a lower LCA/CDCA ratio, indicating an altered product-to-precursor balance in the CDCA-LCA conversion. In contrast, no significant between-group differences were observed in total CA, total DCA, or the DCA/CA ratio. Although several secondary BA-related measures were associated with disease severity, total LCA and the LCA/CDCA ratio were inversely associated with selected histological indices, whereas total CDCA showed no significant associations with the assessed phenotypes. Together, these findings highlighted LCA as a biologically relevant component of the altered BA profile.

Although LCA can signal through several bile acid-responsive receptors (Makishima et al., 1999), the receptor pathway and downstream effectors involved in its mucosal effects during colitis remain incompletely defined. In our *in vitro* epithelial model, LCA supplementation significantly mitigated DSS-induced inflammatory responses. Importantly, targeted inhibition of miR-29a partially weakened the anti-inflammatory effects of LCA. These findings suggest that miR-29a contributes to the epithelial anti-inflammatory response to LCA. Previous studies in hepatic stellate cells have identified FXR as an upstream transcriptional regulator of miR-29a (Li et al., 2011), suggesting one possible receptor-mediated mechanism that remains to be examined in intestinal epithelial cells. Together with the *in vivo* association of miR-29a/b1 deficiency with changes in the gut microbiota and BA profile, these findings support a possible bidirectional host-microbiota-metabolite relationship. MiR-29a/b1 status may influence the intestinal microbial-metabolic environment, while LCA may in turn act partly through miR-29a in epithelial cells. Further studies are required to resolve the relative contributions of miR-29a/b1 status and the inflammatory environment and to define the receptor mechanisms underlying this reciprocal regulation.

Targeted modulation of the gut microbiota-BA axis, particularly restoration of beneficial secondary BA signaling, has therapeutic potential for IBD (Pan et al., 2024). However, systemic administration of secondary BAs such as LCA may be limited by adverse metabolic effects, including systemic toxicity and body weight loss (Ward et al., 2017). Our findings suggest that miR-29a contributes to epithelial responsiveness to microbiota-derived LCA in the inflamed gut and may therefore represent one host factor shaping responses to microbial metabolites. Accordingly, future studies should assess whether mucosal miR-29a status influences responses to BA-targeted therapies or microbiota-oriented nutritional interventions, including probiotic and prebiotic strategies to support BA-metabolizing communities and approaches aimed at restoring LCA signaling. Such integration of microbial, metabolic, and host epigenetic features may help inform future precision nutrition and metabolite-directed strategies for IBD management (Sun et al., 2026). Localized modulation of microbial BA metabolism or intestinally restricted delivery of LCA may also warrant evaluation as approaches to enhance mucosal anti-inflammatory effects while limiting systemic exposure.

Despite these translational perspectives, certain constraints of the present study should be considered. A primary limitation of the current study is that, although we found that miR-29a contributes to the epithelial anti-inflammatory response to LCA, the upstream receptor mechanism remains unresolved. Previous reports of PXR-mediated anti-inflammatory signaling in the intestine (Huang et al., 2018) and FXR-dependent induction of miR-29a in hepatic stellate cells (Li et al., 2011) suggest two receptor mechanisms under certain conditions, whose relevance to intestinal epithelial cells requires receptor-specific pharmacological or genetic testing. Additionally, the acute DSS model and Caco-2 system do not fully reproduce the chronic, relapsing nature of human IBD or complex immune-stromal interactions within the intestinal mucosa. Because microbial metabolites and microRNAs collaboratively orchestrate mucosal immunity, future investigations utilizing intestinal organoids co-cultured with immune cells could help define the broader impact of this epigenetic-metabolic axis on the immune microenvironment. Methodologically, PICRUSt2 inference from 16S rRNA profiles does not directly measure the abundance, transcription, or activity of BA-metabolizing genes such as *bsh* and *hdhA*; shotgun metagenomic and targeted functional analyses are therefore needed to validate the predicted metabolic potential. Finally, the precise temporal sequence linking miR-29a deficiency, dysbiosis, and inflammation remains to be mapped. Longitudinal studies and fecal microbiota transplantation (FMT) applied to germ-free mice will be crucial to establish the exact directionality of this cross-kingdom crosstalk.

## 5 Conclusion

Ultimately, our findings support an association between miR-29a/b1 deficiency and altered gut microbial BA homeostasis during DSS-induced colitis, characterized by greater disease severity, gut microbial dysbiosis, and disruption of the CDCA-LCA balance. *In vitro* data further indicate that epithelial miR-29a contributed to the anti-inflammatory response to LCA. These findings provide an epigenetic-metabolic perspective for understanding intestinal homeostasis, highlighting the role of host microRNAs in shaping epithelial responses to protective microbial metabolic signals.

## Data Availability

The datasets presented in this study are publicly available in the Genome Sequence Archive (GSA) under accession number CRA040687: https://ngdc.cncb.ac.cn/gsa.
