# Supplementary material for: MiR-29a deficiency disrupts gut microbiota-bile acid homeostasis and impairs the anti-inflammatory effects of lithocholic acid in colitis
Source: Front Cell Infect Microbiol. 2026 Sep 17;16:1867553. doi: 10.3389/fcimb.2026.1867553 (PMC13627037; doi:10.3389/fcimb.2026.1867553)
Supplement: Supplementary file 1 [file DataSheet1.pdf]

Supplementary Material

Supplementary Tables

Table S1. Scoring criteria for the Disease Activity Index (DAI).

Score	Weight loss	Stool consistency	Intestinal bleeding
0	None	Normal	Negative hemoccult
1	1–5%	Soft but still formed	Negative hemoccult
2	6–10%	Soft	Positive hemoccult
3	11–18%	Very soft; wet	Blood traces in stool visible
4	>18%	Watery diarrhea	Gross rectal bleeding

Table S2. Scoring system for inflammation-associated histological changes.

Erosion or Ulceration	Oedema	Hyperplasia	Inflammation severity	Crypt damage	Depth of lesion	Score
None	None	Normal	None	None	None	0
< 1 mm	Present	Mild	Slight	Crypt expansion	Mucosa	1
> 1 mm	-	Moderate	Moderate	Basal 1/3 damaged	Submucosa	2
-	-	Severe	Severe	Basal 2/3 damaged	Muscularis	3
-	-	-	None	Crypts lost, surface epithelium present	Serosa	4
-	-	-	-	Crypts and surface epithelium lost	-	5

Table S3. Primers used for quantitative real-time PCR (qPCR).

mmu	Primer sequence (5'-3')
Il6	CACAAGTCCGGGAGGAGAC
	TCCACGATTTCCCAGAGAAC
Il1b	TCGTGCTGTCGGACCCATAT
	GCCCAAGGCCACAGGTATTT
Tnf	CCAAATGGCCTCCCTCTCAT
	GGTGGTTTGCTACGACGTGG
Cxcl1	CTGGGATTCACCTCAAGAACATC
	CAGGGTCAAGGCAAGCCTC
Cxcl2	CCAACCACCAGGCTACAGG
	GCGTCACACTCAAGCTCTG
Ptgs2	TGAGCAACTATTCCAAACCAGC
	GCACGTAGTCTTCGATCACTATC
Nos2	GAGACAGGGAAGTCTGAAGCAC
	CCAGCAGTAGTTGCTCCTCTTC

hsa	Primer sequence (5'-3')
miR-29a	gcgTAGCACCATCTGAAATCGGTTA
IL6	AGACAGCCACTCACCTCTTCAG
	TTCTGCCAGTGCCTCTTTGCTG
IL1b	GCCAGTGAAATGATGGCTTATT
	AGGAGCACTTCATCTGTTTAGG
TNF	GCCCATGTTGTAGCAAACCC
	TGAGGTACAGGCCCTCTGAT
NFKB1	GCAGCACTACTTCTTGACCACC
	TCTGCTCCTGAGCATTGACGTC
RELA	TGAACCGAAACTCTGGCAGCTG
	CATCAGCTTGCGAAAAGGAGCC
CXCL8	GAGAGTGATTGAGAGTGGACCAC
	CACAACCCTCTGCACCCAGTTT
PTGS2	CGGTGAAACTCTGGCTAGACAG
	GCAAACCGTAGATGCTCAGGGA
NOS2	GCTCTACACCTCCAATGTGACC
	CTGCCGAGATTTGAGCCTCATG

Table S4. List and parameters of the 41 targeted bile acids quantified.

Abbr.	Metabolites	Formula	MW
12-KLCA	12-Ketolithocholic Acid	C24H40O4	404.5
23-Nor-DCA	Nor-Desoxycholic Acid	C26H44NNaO7S	498.7
3β-CA	3β-Cholic Acid	C24H38O4	394.5
3β-UDCA	3β-Ursodeoxycholic Acid	C24H34O5	518.6
6,7-DLCA	6,7-Diketolithocholic Acid	C26H43NO5	408.6
7,12-DKLCA	7,12-Diketolithocholic Acid	C26H43NO3	417.6
7-KLCA	7-Ketolithocholic Acid	C24H40O4	392.6
ALCA	Allocholic Acid	C24H40Na2O7S	408.6
CA	Cholic Acid	C24H40O5	408.3
CDCA	Chenodeoxycholic Acid	C24H40O4	392.3
DCA	Deoxycholic Acid	C24H40O4	392.3
DHCA	Dehydrocholic Acid	C26H43NO6	376.6
EALCA	Epiallolithocholic Acid	C24H38O4	390.6
GCA	Glycocholic Acid	C26H43NO6	465.3
GCDCA	Chenodeoxycholic Acid Glycine Conjugate	C26H43NO5	449.3
GCHCA-Na	Glycohyocholic Acid, Sodium Salt	C26H44NNaO6S	392.6
GDCA	Deoxycholic Acid Glycine Conjugate	C26H43NO5	449.3
GHDCA	Glycohyodeoxycholic Acid	C24H40O5	390.6
GLCA	Lithocholic Acid Glycine Conjugate	C26H43NO4	433.3
GUCA-Na	Glycoursocholanic Acid (Sodium Salt)	C26H45NO7S	449.6
GUDCA	Glycoursodeoxycholic Acid	C26H43NO5	449.3
HCA	Hyocholic Acid	C24H40O4	408.6
HDCA	Hyodeoxycholic Acid	C24H40O5	392.6
Iso-LCA	Isolithocholic Acid	C24H40O5	515.7
LCA	Lithocholic Acid	C24H40O3	376.3
M-DCA	Murideoxycholic Acid	C24H40O5	537.7
NCA	23-Norcholic Acid	C24H40O3	408.6
TCA	Taurocholic Acid	C26H45NO7S	515.3
TCDCA	Taurochenodesoxycholic Acid	C26H45NO6S	499.3
TDCA	Taurodeoxycholic Acid	C26H45NO6S	499.3
THCA-Na	Taurohyocholic Acid (Sodium Salt)	C24H36O5	376.6
THDCA	Taurohyodeoxycholic Acid	C24H36O5	408.6
TLCA	Lithocholyltaurine	C26H45NO5S	483.3
TUDCA	Tauroursodeoxycholic Acid	C26H45NO6S	499.3
UCA	Ursocholic Acid	C24H40O5	515.7
UDCA	Ursodeoxycholic Acid	C24H40O4	392.3
α-MCA	α Muricholic Acid	C23H38O5	404.5
α-TMCA-Na	Tauro-α-Muricholic Acid (Sodium Salt)	C24H40O5	465.6
β-MCA	β-Muricholic Acid	C26H45NO7S	402.5
β-TMCA-Na	Tauro-β-Muricholic Acid (Sodium Salt)	C24H40O5	408.6
γ-MCA	γ-Muricholic Acid	C23H38O4	378.6

Notes: For the pool-level analysis, primary BAs comprised α-MCA, UCA, ALCA, CA, CDCA, UDCA, β-MCA, GCA, TCA, α-TMCA-Na, and TCDCA; secondary BAs comprised LCA, HCA, NCA, DCA, HDCA, M-DCA, 12-KLCA, γ-MCA, 7,12-DKLCA, 3β-CA, THDCA, THCA-Na, TLCA, GDCA, TDCA, and TUDCA. Total CDCA was calculated as the sum of CDCA, GCDCA, and TCDCA, total LCA was calculated as the sum of LCA, GLCA, and TLCA.

Supplementary Figures

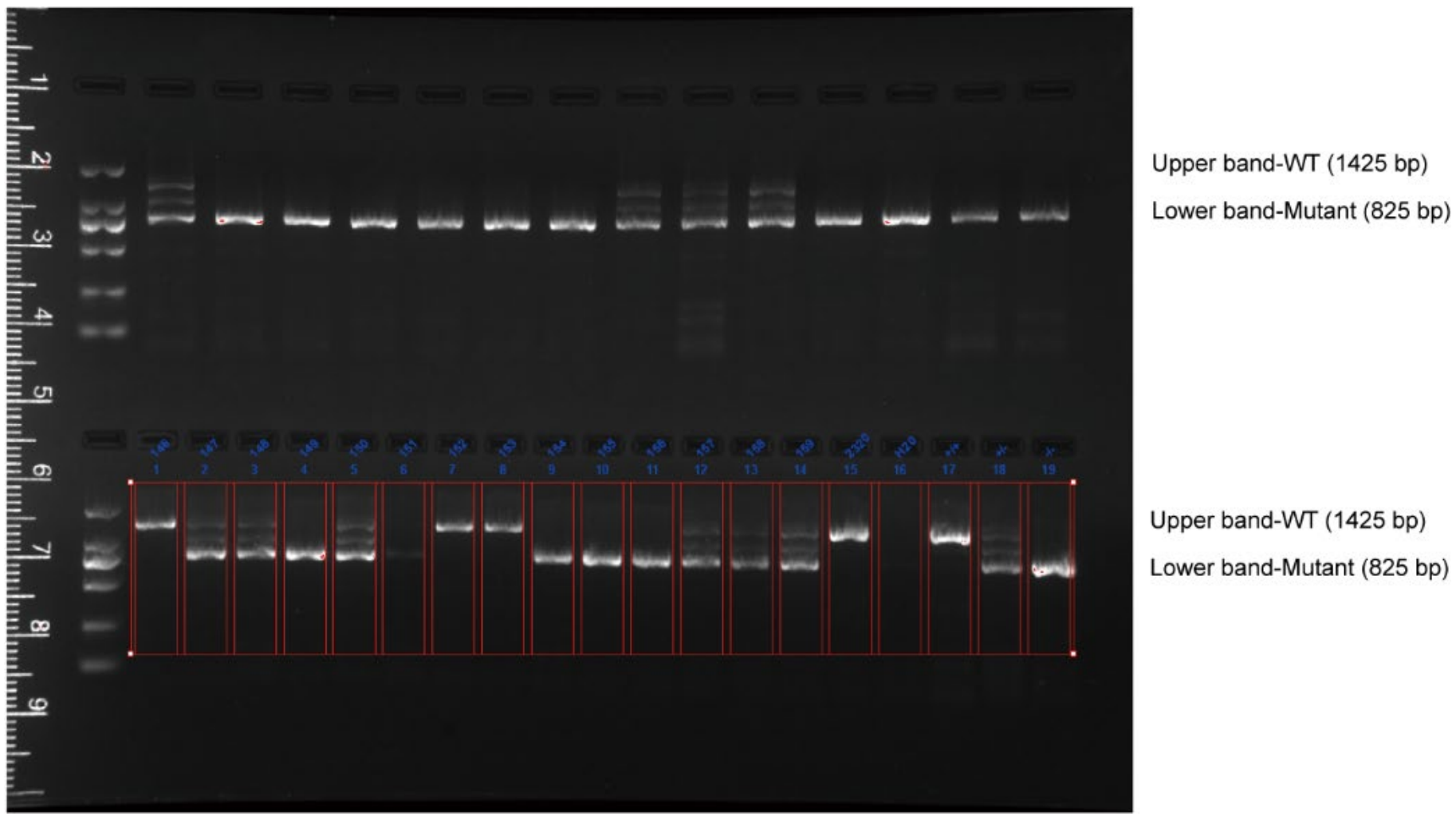

Supplementary Figure 1. Representative PCR genotyping of *Mir29a/Mir29b-1* locus-deficient mice. Bands at 1425 bp and 825 bp correspond to the WT and MUT alleles, respectively. All experimental animals were pre-screened and confirmed by genotyping. WT: wild type; MUT: mutant.

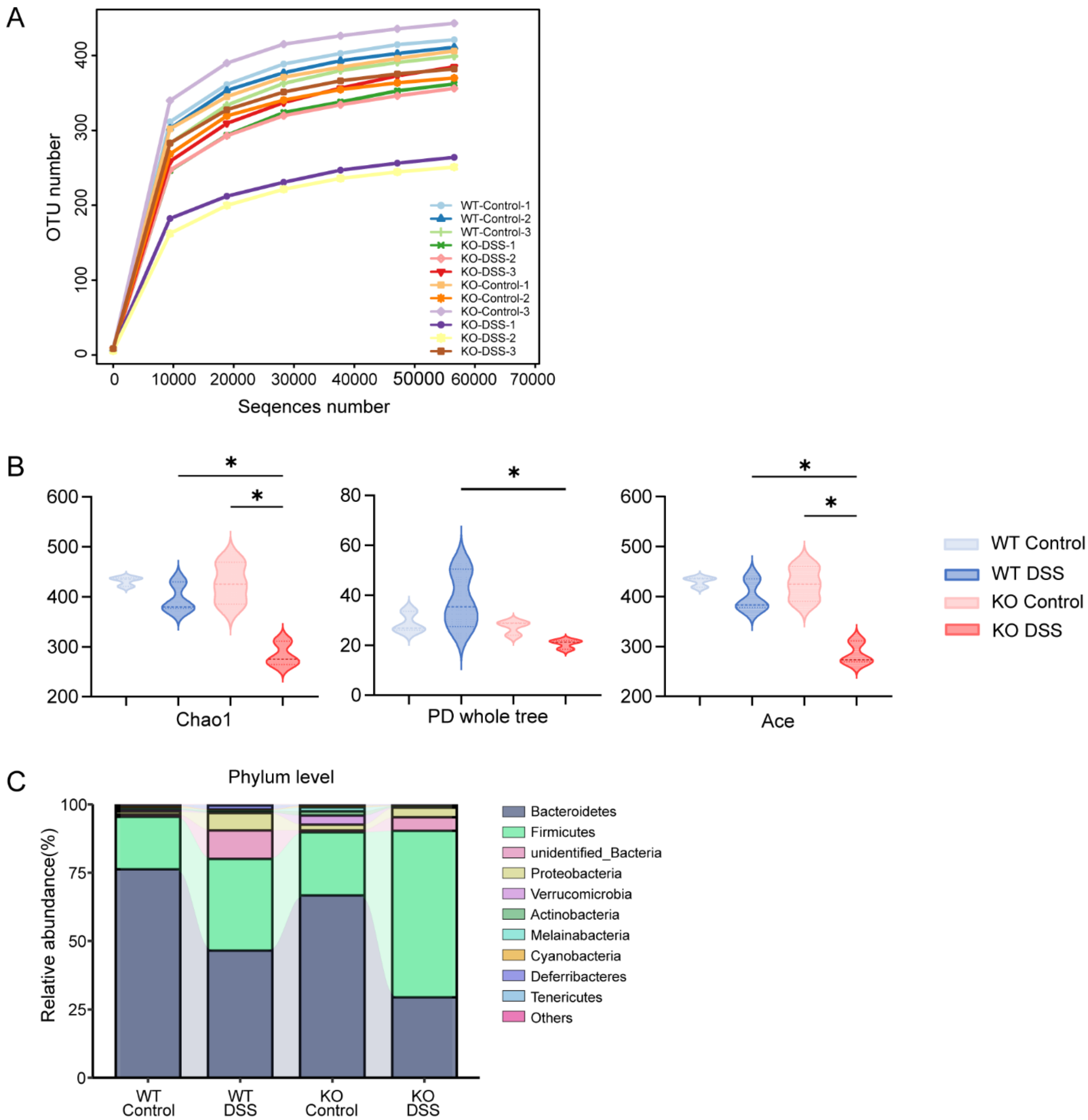

Supplementary Figure 2. Rarefaction curves, alpha diversity and phylum-level taxonomic analysis. **(A)** Rarefaction curves of observed OTUs for individual fecal samples. Each curve represents one mouse. **(B)** Alpha diversity indices, including Chao1, PD_whole_tree, and ACE. **(C)** Relative abundance of gut microbiota at the phylum level. Data are presented as mean ± SD (n = 3 per group). Differences in B were assessed using the Kruskal-Wallis test with Dunn's post hoc test. *$p < 0.05$. OTU: operational taxonomic unit; WT: wild type; KO: knockout; DSS: dextran sulfate sodium.

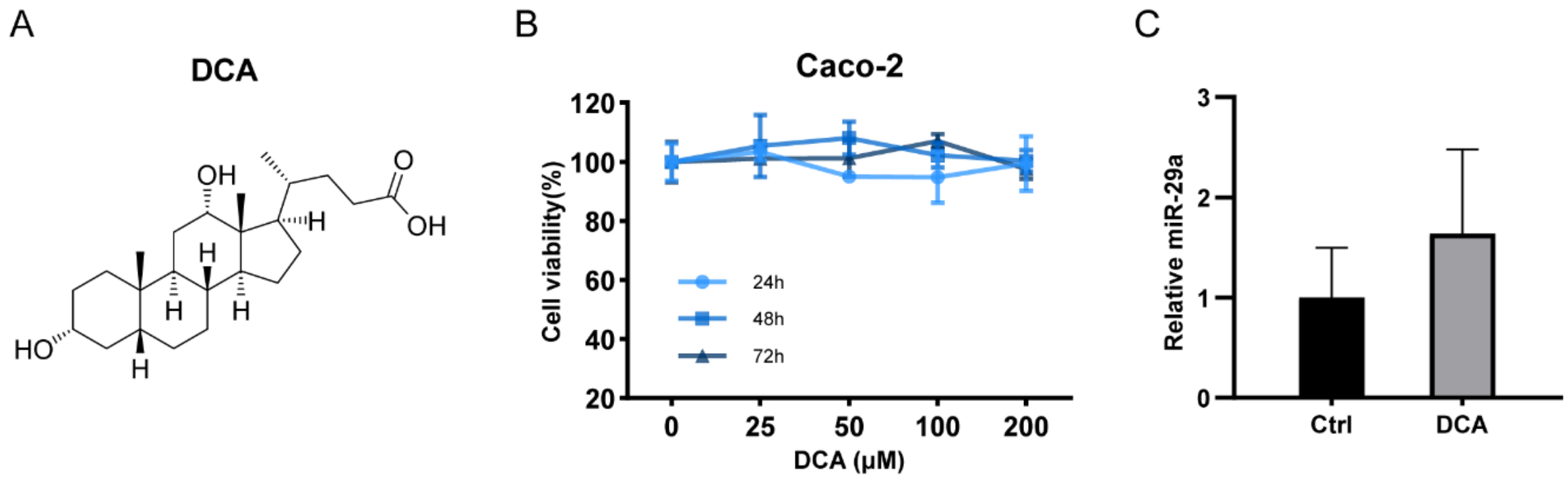

Supplementary Figure 3. Effects of DCA treatment on Caco-2 cells in vitro. **(A)** Chemical structure of DCA. **(B)** Viability of Caco-2 cells was determined via MTS assay after treatment with increasing concentrations of DCA (0, 25, 50, 100 and 200 μM) for the indicated time periods (24, 48 and 72 h). **(C)** Relative expression level of miR-29a in DCA-treated (50 μM, 48 h) Caco-2 cells. MiRNA levels were normalized to *U6*. Data are presented as mean ± SD from 3 independent biological experiments. The comparison in panel C was performed using Student's t-test; $p > 0.05$. DCA: deoxycholic acid.

Supplementary Methods

1. 16S rRNA gene amplification and sequencing

Genomic DNA concentration was quantified using a Qubit fluorometer, and DNA integrity was evaluated by 1% agarose gel electrophoresis, then DNA was diluted to 1 ng/μL with sterile water. PCR was performed in a total volume of 30 μL containing 15 μL of Phusion High-Fidelity PCR Master Mix (New England Biolabs), 0.2 μM each primer, and 10 ng of template DNA. Thermal cycling consisted of an initial denaturation at 98°C for 1 min, followed by 30 cycles at 98°C for 10 s, 50°C for 30 s, and 72°C for 30 s, with a final extension at 72°C for 5 min.

PCR products were examined by 2% agarose gel electrophoresis, pooled at equal concentrations, and purified using the GeneJET Gel Extraction Kit (Thermo Scientific). Sequencing libraries were prepared using the Ion Plus Fragment Library Kit 48 rxns and assessed using a Qubit 2.0 Fluorometer before sequencing.

Single-end reads were demultiplexed according to their barcode sequences, and barcode and primer sequences were removed. Raw reads were quality-filtered using Cutadapt version 1.9.1. Chimeric sequences were identified against the SILVA reference database using the UCHIME algorithm and removed to obtain clean reads. OTU clustering was performed using UPARSE version 7.0.1001. Representative sequences were taxonomically annotated using the mothur algorithm against the SILVA database and aligned using MUSCLE version 3.8.31. Observed species, Chao1, ACE, Shannon, Simpson, Good's coverage, and PD_whole_tree were calculated using QIIME version 1.7.0. Rarefaction curves were generated based on observed OTUs.

2. Microbiome statistical analysis

Exact permutation procedures were used for the PERMANOVA and PERMDISP analyses. Pairwise PERMANOVA was restricted to three prespecified comparisons: WT-Control versus WT-DSS, KO-Control versus KO-DSS, and WT-DSS versus KO-DSS, with Benjamini-Hochberg correction applied to the resulting p values. Genus-level Dunn's post hoc tests were restricted to the same comparisons, with Holm adjustment applied within each genus. For correlation analyses, the p values from all genus-phenotype associations were adjusted together using the Benjamini-Hochberg procedure.
